# Genome-Wide Association Analyses for Fatty Acid Composition in Porcine Muscle and Abdominal Fat Tissues

**DOI:** 10.1371/journal.pone.0065554

**Published:** 2013-06-07

**Authors:** Bin Yang, Wanchang Zhang, Zhiyan Zhang, Yin Fan, Xianhua Xie, Huashui Ai, Junwu Ma, Shijun Xiao, Lusheng Huang, Jun Ren

**Affiliations:** Key Laboratory for Animal Biotechnology of Jiangxi Province and the Ministry of Agriculture of China, Jiangxi Agricultural University, Nanchang, China; University of Queensland, Australia

## Abstract

Fatty acid composition is an important phenotypic trait in pigs as it affects nutritional, technical and sensory quality of pork. Here, we reported a genome-wide association study (GWAS) for fatty acid composition in the *longissimus* muscle and abdominal fat tissues of 591 White Duroc×Erhualian F_2_ animals and in muscle samples of 282 Chinese Sutai pigs. A total of 46 loci surpassing the suggestive significance level were identified on 15 pig chromosomes (SSC) for 12 fatty acids, revealing the complex genetic architecture of fatty acid composition in pigs. Of the 46 loci, 15 on SSC5, 7, 14 and 16 reached the genome-wide significance level. The two most significant SNPs were ss131535508 (*P* = 2.48×10^−25^) at 41.39 Mb on SSC16 for C20∶0 in abdominal fat and ss478935891 (*P* = 3.29×10^−13^) at 121.31 Mb on SSC14 for muscle C18∶0. A meta-analysis of GWAS identified 4 novel loci and enhanced the association strength at 6 loci compared to those evidenced in a single population, suggesting the presence of common underlying variants. The *longissimus* muscle and abdominal fat showed consistent association profiles at most of the identified loci and distinct association signals at several loci. All loci have specific effects on fatty acid composition, except for two loci on SSC4 and SSC7 affecting multiple fatness traits. Several promising candidate genes were found in the neighboring regions of the lead SNPs at the genome-wide significant loci, such as *SCD* for C18∶0 and C16∶1 on SSC14 and *ELOVL7* for C20∶0 on SSC16. The findings provide insights into the molecular basis of fatty acid composition in pigs, and would benefit the final identification of the underlying mutations.

## Introduction

The pig is an important domesticated animal that produce approximate 40% of red meat worldwide [1]. The composition of fatty acids that differ in carbon length and degree of saturation is a crucial factor influencing pork quality and human health. Meat with higher percentage of saturated fatty acid is firmer, while less saturated fat is softer and prone to oxidation and rancidity [2], [3]. High contents of monounsaturated and polyunsaturated fatty acids, especially *n-3* fatty acids in meat are beneficial to cardiovascular health of humans [4], whereas high amount of saturated fatty acids, especially myristic (C14∶0) and palmitic (C16∶0) acids, could increase the risk of coronary heart disease [2].

In humans, abnormal metabolism of fatty acids has been linked to many diseases. For instance, excessive synthesis of fatty acids is a characteristic of many human cancers [5]; reduced polyunsaturated fatty acid composition in skeletal muscle phospholipids is associated with decreased insulin sensitivity [6]. Pigs are much more similar to humans compared to mouse in term of genome structure and biological features [7]. Therefore, elucidating the genetic basis of fatty acid composition in pigs can not only establish novel tools to optimize fatty acid composition of pork, but also gives insights into understand the genomic regulation of fatty acid metabolism in humans.

We and other investigators have detected a number of significant quantitative trait loci (QTL) for fatty acid composition in porcine muscle and fat tissues using genome scans with sparse microsatellite markers [8]–[10]. However, most QTL have confidence intervals of more than 20 Mb, which hampers the identification of underlying genes and variants. Since 2009, high density markers across the pig genome can be genotyped cost-effectively using the Illumina 60 K SNP arrays [11]. Genome-wide association studies (GWAS) have been increasingly conducted to identify genomic regions for a variety of traits including monogenic and quantitative traits in pigs [12], [13]. Moreover, the very recent availability of high quality whole genome draft sequence for pigs [14] would substantially facilitate the characterization of functional genes within a given genomic region. For fatty acid composition in pork, only one very recent GWAS has been reported on muscle samples of an Iberian×Landrace cross [15], and no responsible gene has been characterized. The molecular basis of fatty acid composition in different pig tissues and populations requires further investigations.

In this study, we conducted a conditional GWAS for fatty acid compositions in abdominal fat and *longissimus dorsi* muscle of 591 pigs from a White Duroc×Erhualian F_2_ intercross [8], and in the *longissimus* muscle samples of 282 pigs from a Chinese Sutai half-sib population. A meta-analysis of GWAS was further implemented on the two experimental populations. The results showed genome-scale loci associated with fatty acid compositions in the two tested tissues, and revealed a number of critical regions and several promising candidate genes for follow-up investigations of the underlying genes and variants. The experimental data are available upon the readers’ request.

## Results

### Phenotypic Values

We investigated 12 fatty acids with 14 to 20 carbons that represent the majority (>97%) of total fatty acids across samples (**Table 1**). The fatty acid composition in the White Duroc×Erhualian F_2_ population has been reported in our previous publications [8], [16], [17], while the fatty acid composition in the *longissimus dorsi* muscle samples of Sutai pigs is presented for the first time. The phenotypic values between the two populations were generally comparable despite that they were measured on different platforms (Materials and Methods). The most abundant fatty acid was C18∶1, followed by C16∶0, C18∶0 and C18∶2 in both the *longissimus* muscle and abdominal fat. These fatty acids accounted for the majority (∼90%) of the total fatty acid content. In contrast, arachidonic acid (C20∶4) was the less abundant fatty acid in the tested samples. We also determined heritability estimates of 12 fatty acids. Most of fatty acids have heritability estimates between 0.3 and 0.6, suggesting considerable genetic contribution to fatty acid compositions in muscle and fat tissues (**Table 1**).

**Table 1:** Summary statistics for fatty acid composition in the tested samples^a^.

Trait	F_2_ (Muscle)			F_2_ (Fat)			Sutai (Muscle)		
	N	Mean ± SD	*h* ^2^	N	Mean ± SD	*h* ^2^	N	Mean ± SD	h^2^
Myristic (C14∶0)	589	1.10±0.17	0.39	572	1.10±0.14	0.45	282	1.51±0.66	0.00
Palmitic (C16∶0)	591	23.54±1.32	0.36	572	24.33±1.33	0.47	282	25.69±1.66	0.07
Palmitoleic (C16∶1*n*-7)	591	3.00±0.52	0.39	572	1.74±0.38	0.56	282	3.14±0.90	0.33
Stearic (C18∶0)	591	13.10±1.19	0.37	572	14.55±1.94	0.44	282	13.88±1.64	0.46
Oleic (C18∶1*n*-9)	591	44.38±3.39	0.34	572	41.12±3.58	0.41	282	42.92±4.57	0.17
Linoleic (C18∶2*n*-6)	591	8.92±2.73	0.37	572	12.94±2.76	0.41	282	8.47±3.26	0.49
Linolenic (C18∶3*n*-3)	589	0.19±0.05	0.37	572	0.44±0.11	0.30	189	0.22±0.17	-
Arachidic (C20∶0)	591	0.24±0.07	0.64	572	0.25±0.07	0.45	207	0.30±0.07	0.64
Eicosenoic (C20∶1*n*-9)	591	0.84±0.18	0.56	572	0.91±0.23	0.53	281	1.03±0.24	0.28
Eicosadienoic (C20∶2*n*-6)	590	0.44±0.13	0.33	572	0.62±0.14	0.37	272	0.39±0.14	0.71
Homolonolenic (C20∶3*n*-6)	591	1.41±0.84	0.44	572	0.17±0.05	0.31	281	0.18±0.17	0.10
Arachidonic (C20∶4*n*-6)	479	0.05±0.02	0.28	572	0.08±0.02	0.18	189	0.08±0.07	0.68

a The phenotypes of F_2_ animals genotyped for 183 microsatellite markers have been reported in our previous QTL mapping study [8]. *h* ^2^, heritability estimates.

### Impact of Sample Structure on GWAS

The principle components analysis on the 60K genotype data showed the clear divergence between the F_2_ and Sutai populations (data not shown). We thus performed separate analyses of GWAS on the two populations. The average inflation factors (λ) of the GWAS for all fatty acids in F_2_ and Sutai pigs were 1.10 and 1.06 respectively, indicating that the population structures were properly adjusted and had minor effect on the GWAS results.

### Summarization of the GWAS Results

We identified a total of 46 loci on 15 chromosomes that satisfied suggestive significance for 12 fatty acids (**Table 2** **and Table S1**), reflecting the complexity of genetic regulation on fatty acid metabolism in fat and muscle tissues of pigs. Of these loci, 13 out of 37 loci identified in F_2_ pigs and 2 out of 9 loci in Sutai pigs surpassed the genome-wide significance level (**Table 2**). The 15 genome-wide significant loci included one locus on SSC5 for muscle C20∶0; 9 loci on SSC7 for C18∶1, C18∶2, C18∶3, C20∶1 and C20∶3 in abdominal fat and C18∶3, C20∶1 and C20∶2 in muscle; 3 loci on SSC14 for C18∶0 in both fat and muscle tissues and C16∶1 in muscle; and 3 loci on SSC16 for C20∶0 in both fat and muscle tissues. These loci explain 4.8–34.8% of total phenotypic variance (**Table 2**). The most significant SNP across all traits was ss131535508 (*P* = 2.48×10^−25^) at 41.39 Mb on SSC16, which accounted for 34.8% of phenotypic variance in abdominal fat C20∶0 content of F_2_ animals. The second top signal was ss478935891 (*P* = 3.29×10^−13^) at 121.31 Mb on SSC14, which explained 18.4% of phenotypic variance in muscle C18∶0 of Sutai pigs.

**Table 2:** Genome-wide significant loci identified by GWAS for fatty acid composition in White Duroc×Erhualian F_2_ animals and Sutai pigs^a^.

Chromosome	Trait	Population	Tissue b	N_snp_ c	Top SNP	Position (bp)	*P*-value	Var (%) d	Candidate gene
5	C20∶0	F_2_	LD	3	ss131292619	77556266	1.96E-07	4.8	*ADIPOR2, ABCD2*
7	C18∶3	F_2_	LD	81	ss107806758	35177641	8.42E-10	12.5	*PPARD*, *HMGA1*
	C18∶3	F_2_	AF	1	ss107837325	34803564	1.23E-06	10.9	
	C20∶3	F_2_	AF	75	ss131344094	35251345	5.88E-10	13.9	
	C18∶1	F_2_	AF	34	ss107837325	34803564	5.07E-08	12.7	
	C18∶2	F_2_	AF	23	ss107837325	34803564	1.08E-07	7.0	
	C20∶2	F_2_	LD	2	ss107837325	34803564	6.94E-07	7.2	
	C20∶1	F_2_	AF	75	ss131351882	52184508	2.39E-11	29.9	*ACSBG1*
	C20∶1	F_2_	LD	30	ss131351882	52184508	1.30E-10	20.6	
14	C18∶0	Sutai	LD	24	ss478935891	121305916	3.29E-13	18.4	*SCD*
	C18∶0	F_2_	LD	22	ss478935891	121305916	7.99E-10	9.1	
	C16∶1	F_2_	LD	1	ss131499825	121330920	7.29E-07	6.8	
16	C20∶0	F_2_	AF	121	ss131535508	41393886	2.48E-25	34.8	*ELOVL7*
	C20∶0	F_2_	LD	53	ss131535508	41393886	6.26E-23	31.4	
	C20∶0	Sutai	LD	34	ss131535602	42280991	5.38E-10	26.1	

a The significant loci identified in the meta-analyses are listed in Table S1.

b LD, the *longissimus dorsi* muscle; AF, abdominal fat.

c the number of SNP that surpassed the suggestive significant level at the first round of GWAS.

d Phenotypic variance explained by the top SNPs.

### Comparison of the GWAS Loci for Fatty Acid Composition in Muscle and Fat Tissues

In the F_2_ population, 4 fatty acids including C18∶1, C18∶3, C20∶0 and C20∶1 shared 1, 37, 98 and 59 SNPs surpassing the suggestive significance (*P*<2.53×10^−5^) across muscle and abdominal tissues, respectively. All shared SNPs in the two tissues have the same directional effects on fatty acids (**Table S2**). Moreover, the Pearson correlation between association strength of shared SNPs across the two tissues was highly significant (*P*<10^−16^), highlighting the conservative genetic architecture of C18∶1, C18∶3, C20∶0 and C20∶1 in the two tissues. On the other hand, tissue specific loci were identified for the other fatty acids. For instance, the locus around 121.31 Mb on SSC14 was only significantly associated with muscle C16∶1 and C18∶0, whereas the loci at 89.04 Mb on SSC4 and at 34.80 Mb on SSC7 had specific effect on abdominal fat C18∶2 (**Table 2** **and Table S1**). These observations suggest the existence of both tissue conservative and specific determinants contributing to fatty acid composition in pigs.

### Common and Specific Loci in the Two Studied Populations

**Figure 1:**
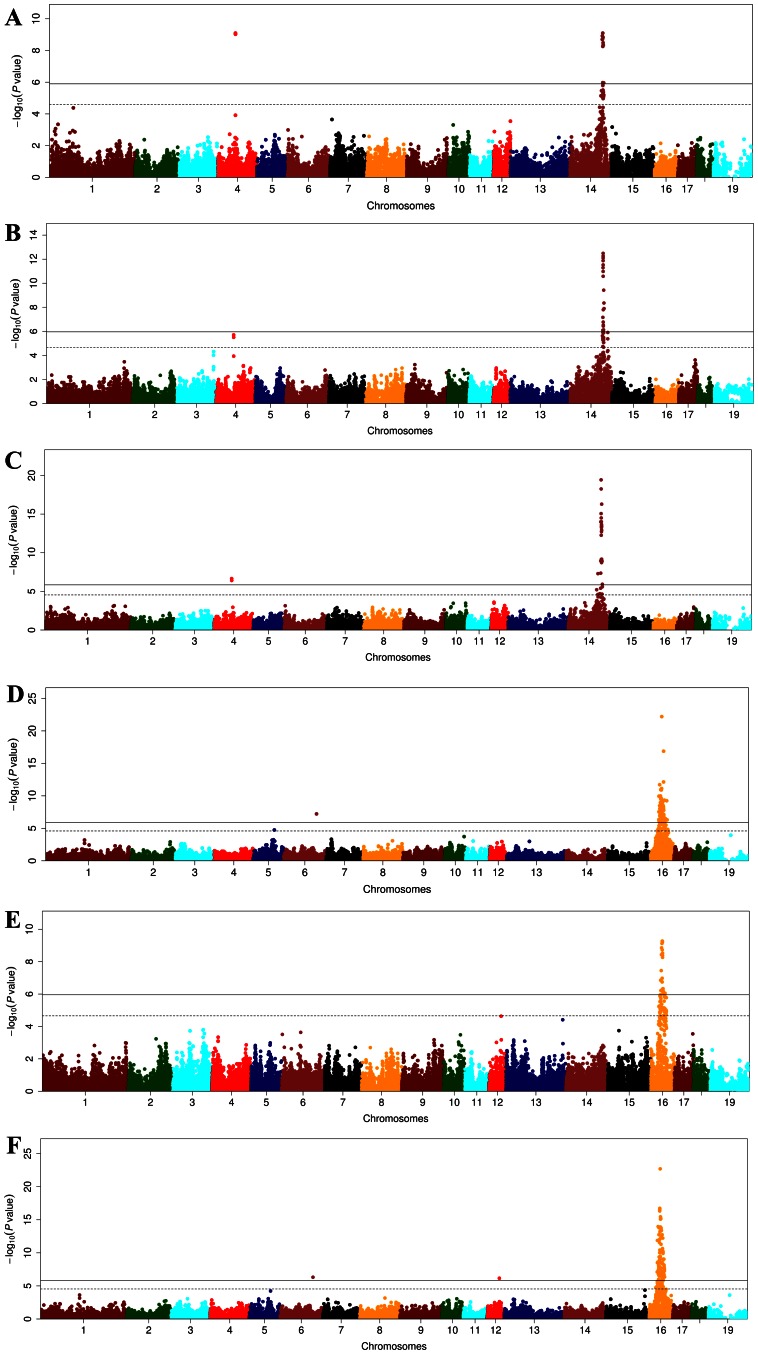
Manhattan plots for the analyses of muscle C18∶0 and C20∶0. (A–C) The first round of GWAS for C18∶0 in F_2_ (A) and Sutai (B) pigs and the meta-analysis of F_2_ and Sutai samples (C). (D–F) The first round of GWAS for C20∶0 in F_2_ (D), Sutai (E) and the meta-analysis of F_2_ and Sutai samples (F). In the Manhattan plots, negative log_10_ *P* values of the qualified SNPs were plotted against their genomic positions. The SNPs on different chromosomes are denoted by different colors. The solid and dashed lines indicate the 5% genome-wide and suggestive Bonferroni-corrected thresholds, respectively.

Only 9 loci surpassing suggestive significance were detected in Sutai pigs, which is much less than the 37 loci identified in the F_2_ cross. This is due to the smaller sample size of Sutai pigs compared with F_2_ animals in this study. Of note, 3 loci were consistently detected in Sutai and F_2_ pigs. Both populations shared the same peak SNP (ss478935891) at 121.31 Mb on SSC14 for C18∶0 in muscle (**Table 2** **and** **Figure 1**). The top SNP (ss131535508) at 41.39 Mb on SSC16 for C20∶0 in both muscle and abdominal fat in the F_2_ cross was only 1.5 Mb away from the strongest SNP (ss131535602) at 42.28 Mb for the same fatty acid in Sutai pigs **(** **Table 2** **and** **Figure 1** **)**. Moreover, both populations showed significant association with muscle C20∶1 around ss131352578 at 53.37 Mb on SSC7 **(Table S1)**. The shared GWAS signals suggest that the common underlying variants cause the above-mentioned QTL effects on C18∶0, C20∶0 and C20∶1 in both two populations. The other loci were identified in either Sutai or F_2_ pigs and were thus considered population specific loci.

### Novel Loci Detected by a Meta-analysis of GWAS

A meta-analysis of GWAS for muscle fatty acid composition allowed us to detect 4 novel loci comprising two for C16∶1 on SSC3 and SSC5, one for C18∶0 on SSC4 and one for C18∶1 on SSC15 by combining the *P*-values of GWAS results from the two populations (**Table S2**). Moreover, the association statistics were increased at 6 loci compared to those evidenced in a single population. These loci include the locus for muscle C18∶1 on SSC4, for muscle C20∶1 on SSC7, for muscle C16∶1, C18∶0 and C18∶1 on SSC14, and for muscle C20∶0 on SSC16 (**Figure 1** **and** **Table S1**). These ‘meta-enforced’ loci are likely caused by common underlying variants. The finding indicates that a meta-analysis of GWAS using more samples would contribute to discovering more loci with moderate effects that likely remain unexplored due to the limited samples in the current populations.

## Discussion

### GWAS Versus Traditional QTL Mapping

Compared to our previous finding of 63 significant QTL [8], less significant loci (n = 37) were detected by the GWAS in the same F_2_ cross. This may be due to the stringent Bonferroni-corrected threshold and different model of GWAS. Here the Bonferroni correction of the multiple tests treated all qualified SNPs as independent loci. The conservative threshold can reduce false discovery rate but also decrease the power to detect loci with moderate or small effects. Moreover, only additive SNP effects were included in the mixed linear GWAS model in contrast to the additive and dominant effects considered in the QTL model. However, 23 out of 37 (62.2%) GWAS loci (**Table S1**) confirmed the previously detected QTL. For example, the QTL on SSC4 for C18∶1, C18∶2 and C20∶2, and the QTL on SSC7 for C18∶1, C18∶2, C18∶3, C20∶1, C20∶2, C20∶3 and C20∶4 were replicated in this study [8].

In addition to the confirmed loci, we detected 14 novel loci (**Table S1**). The most remarkable finding is the major locus around 121.31 Mb on SSC14 for muscle C18∶0, C18∶1 and C16∶1 (**Table 1**). This locus was not identified in the previous QTL mapping study [8]. A reasonable explanation for the discrepancy is that the underlying mutation(s) at the SSC14 locus is segregating within founder breeds of the F_2_ cross. The QTL interval mapping was conducted with the assumption that the causal variant is alternatively fixed in the two founder breeds of the F_2_ cross, thereby reducing the power to detect the SSC14 locus. In contrast, GWAS exploits the linkage disequilibrium (LD) between markers and causal variants, which would efficiently detect the variants segregating within founder breeds of F_2_ populations. Indeed, the lead SNP (ss478935891) at the SSC14 locus is segregating in the two White Duroc founder boars. This observation highlights the advantage of GWAS over the traditional linkage analyses of QTL.

### Allelic Heterogeneity

Allelic heterogeneity, i.e., more than one independent variants within a gene or region contributing to the traits of interest, has often been observed in human GWAS [18]. In this study, we conducted a conditional GWAS (Material and Methods) that is capable of detecting loci with allelic heterogeneity. We observed only one example of allelic heterogeneity. Three SNPs including ss131351882 (52.18 Mb), ss131352160 (52.53 Mb) and ss107804785 (53.10 Mb) on SSC7 were evidenced to be independently associated with C20∶1 in abdominal fat (**Figure S1**). For the other loci, all surrounding significant SNPs disappeared when adjusting for the lead SNP (data not shown). It is thus likely that only one causal variant underlies each significant locus. However, additional large samples and higher density markers are needed to address if allelic heterogeneity is rare or common in pigs.

### Impact of Adjusting for Fat Deposit Trait on GWAS Results

Covariates in statistical models have profound impact on the genetic mapping results [16], [19], [20]. Fatness traits, such as backfat thickness, are biologically correlated with fatty acid contents. In this study, we compared association statistics (-log10 *P*-value) at the identified loci under the models with or without average backfat thickness as a covariate (**Figures S2, S3 and S4**). Notably, the significant SNPs around ss107837325 at 34.80 Mb on SSC7 for C18∶1, C18∶2, C18∶3 and C20∶2 in both tissues vanished when controlling for backfat thickness (**Figure S2 and S3**). The region showed the strongest effect on fat deposition traits in the tested populations across the genome [21]. Therefore, we believe that the SSC7 QTL effect on fatty acid composition is indirectly caused by the underlying mutation for fat deposition. Moreover, the significant locus for C18∶2 and C20∶2 on SSC4 defined by ss131270860 at 88.39 Mb and ss120030566 at 91.70 Mb disappeared after correcting for backfat thickness. The region is also a major QTL for fatness traits [21] and likely causes the indirect effect on fatty acid composition. In contrast, the association statistics of loci elsewhere were not affected by the adjustment of backfat thickness (**Figures S2, S3 and S4**). Thereby, these loci are likely directly involved in regulation of fatty acid metabolism.

### Plausible Candidate Genes at the Identified Loci

To identify interesting candidate genes, we searched annotated genes with functional relevance to fatty acid or lipid metabolism in an interval of 10 Mb centered at the top SNP at each significant locus. The large interval was adopted as high LD extents were expected in the current experimental populations. Notable, we found several strong candidate genes at the genome-wise significant loci.

**Figure 2:**
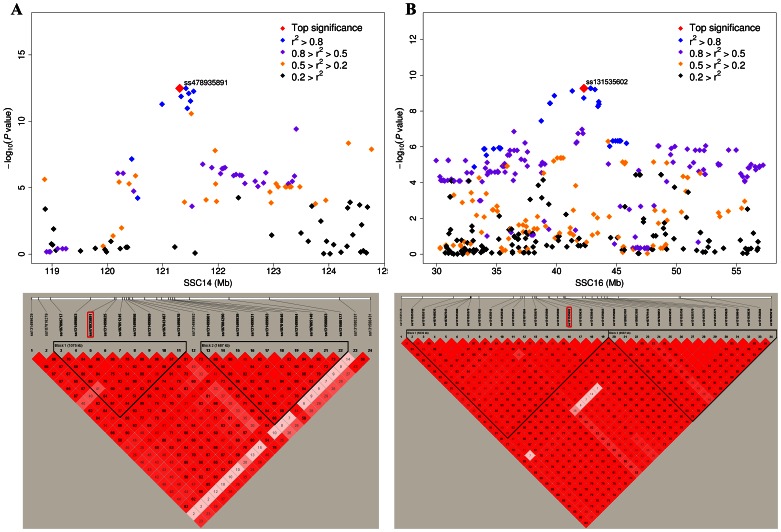
Regional plots of the two major loci on SSC14 and SSC16. Results are shown for muscle C18∶0 on SSC14 (A) and for muscle C20∶0 on SSC16 (B) in Sutai pigs. In the upper panels, the blue diamonds represent the lead SNPs. Different levels of linkage disequilibrium (LD) between the lead SNPs and surrounding SNPs are indicated in different colors. In the lower panels, LD heat maps of SNPs in the two regions are depicted. The top SNPs are highlighted by red rectangles.

On chromosome 14, a number of SNPs around 121.31 Mb were significantly associated with C18∶0, C18∶1 and C16∶1 at the first round of GWAS. This region is concordant with the recently reported locus associated with C18∶0, C18∶1 and melting point of fat reported in a purebred Duroc population [22]. The association was observed in muscle but not in abdominal fat samples, thereby suggesting a tissue-specific regulation of the locus. Moreover, the lead SNP for C18∶0 in the region was ss478935891 in both F_2_ and Sutai pigs, indicating that a common variant causes the QTL effect on the two populations (**Figure 1**). We defined the most likely region of the major locus by LOD dropoff 2 from the strongest SNP. In Sutai pigs, the critical region is only ∼ 500 kb (120.98 Mb –121.50 Mb) and constitutes a LD block (**Figure 2**). A close examination on the critical region revealed that the stearoyl-CoA desaturase (*SCD*) gene at 121.10 Mb is a promising candidate of the locus. SCD is a rate-limited enzyme in the oxidation of fatty acids and preferably catalyze the reaction of stearic acid (C18∶0) and palmitic acid (C16∶0) to oleic acid (C18∶1) and palmitoleic acid (C16∶1) [23]. Two SNPs in the promoter region of *SCD* have been shown to be strongly associated with intramuscular C18∶0 contents (*P* = 6.7×10^−16^) in Duroc pigs [24]. Further investigation in the *SCD* region is thus warranted to identify causal variant(s) for C18∶0, C18∶0 and C16∶0 in our samples. It should be noted that one SNP (ss478943160) on SSC4 showed the same association strength for muscle C18∶0 to the top SNP on SSC14 (**Figure 1**). The two SNPs were in complete linkage disequilibrium (*r^2^* = 1), and no recombination event was observed for them in the F_2_ pedigree. Therefore, we conclude that ss478943160 should be located in the *SCD* region on SSC14 rather than SSC4.

Chromosome 16 encompasses a major locus for C20∶0 in both abdominal fat and muscle samples of F_2_ animals and as well as muscle samples of Sutai pigs. A cluster of SNPs showed strong association signals at the first-round GWAS and the top signal was observed for ss131535508 (41.39 Mb, *P* = 2.48×10^−25^) in F_2_ pigs and for ss131535602 (42.28 Mb, *P* = 5.38×10^−10^) in Sutai pigs on this chromosome (**Table 2**). This region was also evidenced to be associated with intramuscular C20∶1/C20∶0 ratio although at a lower significance level (*P* = 1.5×10^−6^) in an Iberian×Landrace cross [15]. In the F_2_ population, the peak SNP was not in high LD (r^2^>0.8) with any surrounding SNP. In contrast, the lead SNP in Sutai pigs was in high LD with 10 SNPs that defined a confidence region of 4.84 Mb (38.71–43.53 Mb) (**Figure 2**). Within this region, a strong candidate gene at 42.50 Mb, namely *ELOVL7*, is proximal to the peak SNP in Sutai pigs. The *ELOVL7* gene is involved in the elongation of very long-chain fatty acids including C18∶0 and C20∶0 [25]. It is thus worthwhile to perform further investigation on the *ELOVL7* gene to verify its effect on long-chain fatty acids.

Chromosome 7 harbors a QTL-enriched region around 27.13–35.17 Mb. The region is significantly associated with diverse phenotypic traits related to fat deposition, growth and carcass length [21]. Therefore, we speculate that the underlying gene(s) is most likely a global regulator of multiple biological processes, such as *PPARD* and *HMGA1* in the region, rather than a determinant specific for fatty acid metabolism. We have previously identified a causative mutation in *PPARD* for ear size of pigs [26]. Further investigations are required to clarify additional causal variants for the major multifaceted QTL. Another region (50.84–56.22 Mb) on SSC7 is significantly associated with C20∶1 in both abdominal fat and muscle, and with C20∶2 and C20∶4 in abdominal fat of F_2_ pigs at the suggestive level. The top SNP is ss131351882 at 52.18 Mb for C20∶1 in abdominal fat. *ACSBG1* at 53.30 Mb adjacent to the SNP appears to be an interesting candidate gene as it encodes an acyl-CoA synthetase that activates diverse saturated, monounsaturated and polyunsaturated long-chain fatty acids for both synthesis of cellular lipids and degradation through beta-oxidation [27].

On chromosome 5, the loci around 69.37–77.56 Mb were associated with C16∶0 in muscle and C20∶0 in both muscle and abdominal fat. The strongest association was observed between ss131292619 at 77.56 Mb (*P* = 1.96×10^−7^) and muscle C20∶0. *ADIPOR2* and *ABCD2* related to fatty acid or lipid metabolism were found in this region according to the functional annotations by DAVID (http://david.abcc.ncifcrf.gov/).

Interesting candidate genes were also found at several suggestive loci that are consistent with the very recent GWAS report in the Iberian×Landrace cross [13]. *ALDH9A1* [28] and *HSD17B7* [29] that are related to fatty acid metabolism or lipid syntheses reside in the SSC4∶88.39–91.70 Mb region for muscle C20∶2. The top signal for muscle C16∶1 was ss131376859 (*P* = 2.18×10^−6^) at 124.80 Mb on SSC8. Two plausible candidate genes, *ELOVL6* and *MTTP*, are adjacent to this SNP. *ELOVL6* is directly involved in the metabolism of C16∶1 and has been implicated in human obesity related insulin resistance [28]. A *MTTP* missense mutation shows strong association with fatty acid profile in pigs [23]. On chromosome 9, the peak SNP (ss131407752, *P* = 6.27×10^−6^) was associated with muscle C20∶4 in Sutai pigs. Interestingly, *PTGS2* and *PLA2G4A* that are directly involved in metabolism of arachidonic acids (C20∶4) are found in the vicinity of the SNP. Moreover, *PCTP* and *ACACA* have been investigated as candidate genes for the locus on SSC12 where ss107827572 at 41.56 Mb (*P* = 1.79×10^−6^) was associated with muscle C14∶0 of F_2_ pigs in this study.

Altogether, we found several promising candidate genes for fatty acid composition at the identified loci. However, GWAS can not directly identify the causal mutations [29]. Additional studies including fine mapping, functional validation [30] and integrative analyses of intermediate molecule like mRNA expression profiles [31], [32] are needed for further elucidation of the variants underlying the fatty acid composition traits in this study.

### Conclusions

We performed the conditional and meta-analysis of GWAS for 12 fatty acid compositions in fat and muscle tissues from two pig populations. A total of 50 loci on 15 chromosomes surpassed the suggestive significance level, highlighting the complex biological mechanism for fatty acid composition in pig muscle and fat tissues. The two tissues show consistent association profiles at most of the identified loci and distinct association signals at several loci. Three loci have common effects and the other loci have independent effects on the two populations. All significant loci directly influence the metabolism of fatty acids, except that the effects of two loci on SSC4 and SSC7 are indirectly caused by fat deposition. Several promising candidate genes were found in the neighboring regions of the lead SNPs, such as *SCD* for C18∶0 on SSC14 and *ELOVL7* for C20∶0 on SSC16. Our findings provide novel insights into the genetic architecture of fatty acid composition in pigs, and paved the sound road to identify causal variants especially for the major loci on SSC14 and SSC16.

## Materials and Methods

### Ethics Statement

All the procedures involving animals are in compliance with the care and use guidelines of experimental animals established by the Ministry of Agriculture of China. The ethics committee of Jiangxi Agricultural University specifically approved this study.

### Animals and Phenotypes

Experimental animals were from a White Duroc×Erhualian F_2_ cross and a Sutai half-sib population. The F_2_ cross comprises 1912 F_2_ pigs derived from 2 White Duroc founder boars and 17 Chinese Erhualian founder sows (A sub-population of Chinese Taihu pigs) in 6 batches. This population had been employed to detect QTL for a wide variety of traits including fatty acid composition as described in our previous publication [8]. Sutai is a Chinese synthetic pig line that was originally generated from Chinese Taihu and Duroc pigs, and the current Sutai population is developed by over 18-generation of artificial selection. A total of 282 Sutai pigs from 5 sire and 60 dams were used in this study. In the two populations, all piglets were weaned at day 46 and males were castrated at day 90. All fattening pigs were raised under a consistent indoor condition and were fed with ad libitum diet containing 16% crude protein, 3100 kJ digestible energy and 0.78% lysine in the experimental farm of Jiangxi Agricultural University (China), and were slaughtered at the age of around 240 days.

Fatty acid composition traits were measured on *longissimus dorsi* and abdominal fat tissues of 591 F_2_ pigs, and on *longissimus dorsi* samples of 282 Sutai pigs. Muscle between the third and fourth rib and abdominal fat at ventral midline were collected from each animal within 30 min post-mortem, and then stored at −20°C. The total lipid was extracted according to the protocol originally described by Folch *et al*. (1957) using 3∶1 chloroform-methanol solution [33]. About 2 mg obtained lipid was re-dissolved in 2-ml of n-hexane and 1 ml of KOH (0.4 M) for saponification and methylation. The obtained fatty acid methyl esters of F_2_ and Sutai samples were measured using GC2010 gas chromatographer (Shimadzu) and GC6890N (Agilent Technologies, USA), respectively. Each fatty acid was quantified and shown as a percentage of total fatty acids.

### Genotypes and Quality Control

Genomic DNA was extracted from ear tissue of each animal using a standard phenol/chloroform method. A total of 1020 animals from the F_2_ cross and all 282 Sutai pigs were genotyped for 62163 SNPs using the Illumina PorcineSNP60 BeadChip according to the manufacture’s protocol. The quality control (QC) procedures were carried out using Plink v 1.07 software [34], and the same QC criteria were applied on the SNP data from the two populations. Briefly, animals with call rate >0.9 and Mendelian error rate <0.05, and SNP with call rate >0.9, minor allele frequency >0.05, *P* values >10^−6^ for the Hardy-Weinberg equilibrium test and Mendelian error rate <0.1 were included. A final set of 39454 and 45308 SNPs on 591 F_2_ and 282 Sutai pigs were respectively used for subsequent analyses.

### Statistical Analysis

The heritability of a given trait was estimated using the *polygenic* function of GenABEL v1.7 [35]. The associations between SNPs and phenotypic values were evaluated using a mixed model based score test [35]. This method accounted for population structure by fitting the covariance among individuals inferred from high density SNP data. The GWAS were conducted by *polygenic* followed by *mmscore* function of GenABEL v 1.7 [35]. Sex and batch were fitted as fixed effects. At each conditional step, GWAS was conducted controlling for on the peak SNP identified in the previous round scan by iteratively calling the *polygenic* and *mmscore* function in GenABEL until no SNP satisfied the suggestive significance threshold. The multi-locus conditional approach is similar to that described in [36]. The nominal *P*-values were used to represent the association strength between SNPs and phenotypes. The Bonferroni corrected thresholds of 1.3×10^−6^ (0.05/39454) and 1.1×10^−6^ (0.05/45308) were adopted for the 5% genome-wide significance in the F_2_ and Sutai populations, respectively. For suggestive significance, we used the *P*-value thresholds of 2.5×10^−5^ (1/39454) and 2.2×10^−5^ (1/45308), which allowed one false positive signature in one genome scan. The phenotypic variance explained by the top SNPs was estimated by (V_reduce_–V_full_)/V_reduce_, where V_full_ and V_reduce_ are residual variances of models for association analysis with and without SNP term, respectively. For the meta-analysis of GWAS, we used a Z-score approach that combined *P*-values and effects of a common set of 34495 SNPs in both F_2_ and Sutai pigs by employing METAL [37]. Significant SNPs at a distance of more than 10 Mb were considered different loci.

### Annotation of Candidate Genes

The porcine genome assembly 10.2 (http://www.animalgenome.org/repository/pig/Genome_build_10.2_mappings/) was retrieved to characterize candidate genes in targeted regions. The Ensemble Biomart (http://www.biomart.org) online tool was used to find annotated genes within a specific region. DAVID (http://david.abcc.ncifcrf.gov/) was employed to define the function of annotated genes [38]. Linkage disequilibrium measures between SNPs were calculated by Plink v 1.07 software [34].

## Supporting Information

Figure S1 **Conditional GWAS results for C20∶1 in abdominal fat.** From top to bottom panels, the Manhattan plots for the first to fourth round of conditional GWAS are depicted. Multiple independent significant associations were evidenced in the same region on SSC7.
(TIF)

Figure S2 **Comparison of the first round of GWAS results for fatty acid composition in abdominal fat before and after adjusting for backfat thickness in F_2_ animals.** Fatty acid traits are shown under figures in each panel. The panels at the left side show the results from the model without a covariate of backfat thickness, and the right panels represent the results after adjusting for backfat thickness.
(TIF)

Figure S3 **Comparison of the first round of GWAS results for muscle fat fatty acids with or without controlling for backfat thickness in F_2_ animals.** Traits are shown under figures in each panel. The panels at the left side show the results from the model without a covariate of backfat thickness, and the right panels represent the results after correcting for backfat thickness.
(TIF)

Figure S4 **Comparison of the first round of GWAS results for muscle fat fatty acids with or without adjusting for backfat thickness in Sutai pigs.** Traits are shown under figures in each panel. The panels at the left side show the results from the model without a covariate of backfat thickness, and the right panels represent the results after adjusting for backfat thickness.
(TIF)

Table S1 **All loci surpassing the suggestive significance level for fatty acid composition identified in this study.**
(DOC)

Table S2 **Significant SNPs for C18∶1, C18∶3, C20∶0 and C20∶1 across muscle and abdominal fat tissues in the F_2_ population.**
(DOC)
