# Supplementary figures and images for: Genome-Wide Association Analyses for Fatty Acid Composition in Porcine Muscle and Abdominal Fat Tissues
Source: PLoS One. 2013 Jun 7;8(6):e65554. doi: 10.1371/journal.pone.0065554 (PMC3676363; doi:10.1371/journal.pone.0065554)

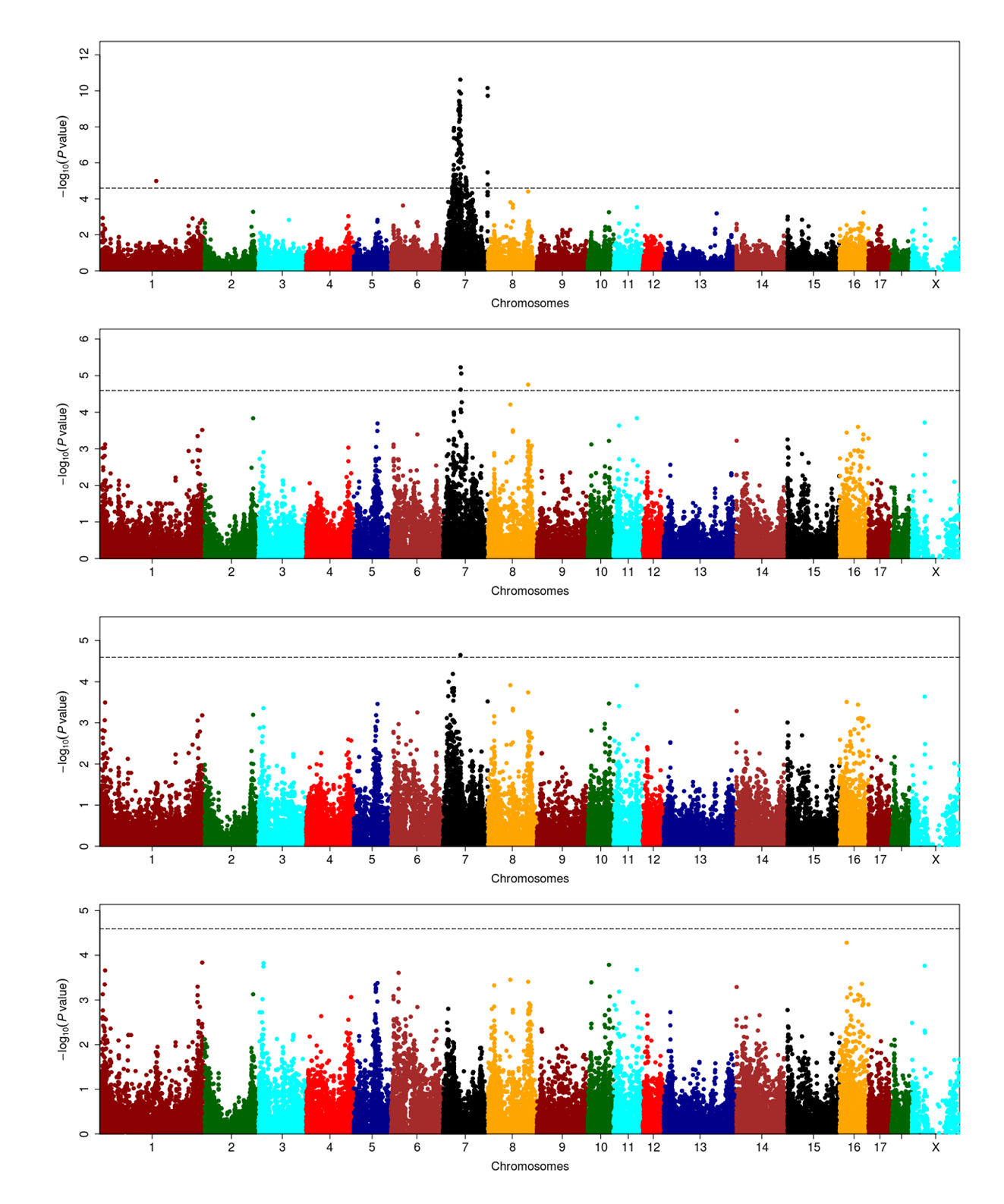

Supplement: Figure S1 — Conditional GWAS results for C20∶1 in abdominal fat. From top to bottom panels, the Manhattan plots for the first to fourth round of conditional GWAS are depicted. Multiple independent significant associations were evidenced in the same region on SSC7. (TIF) [file pone.0065554.s001.tif]

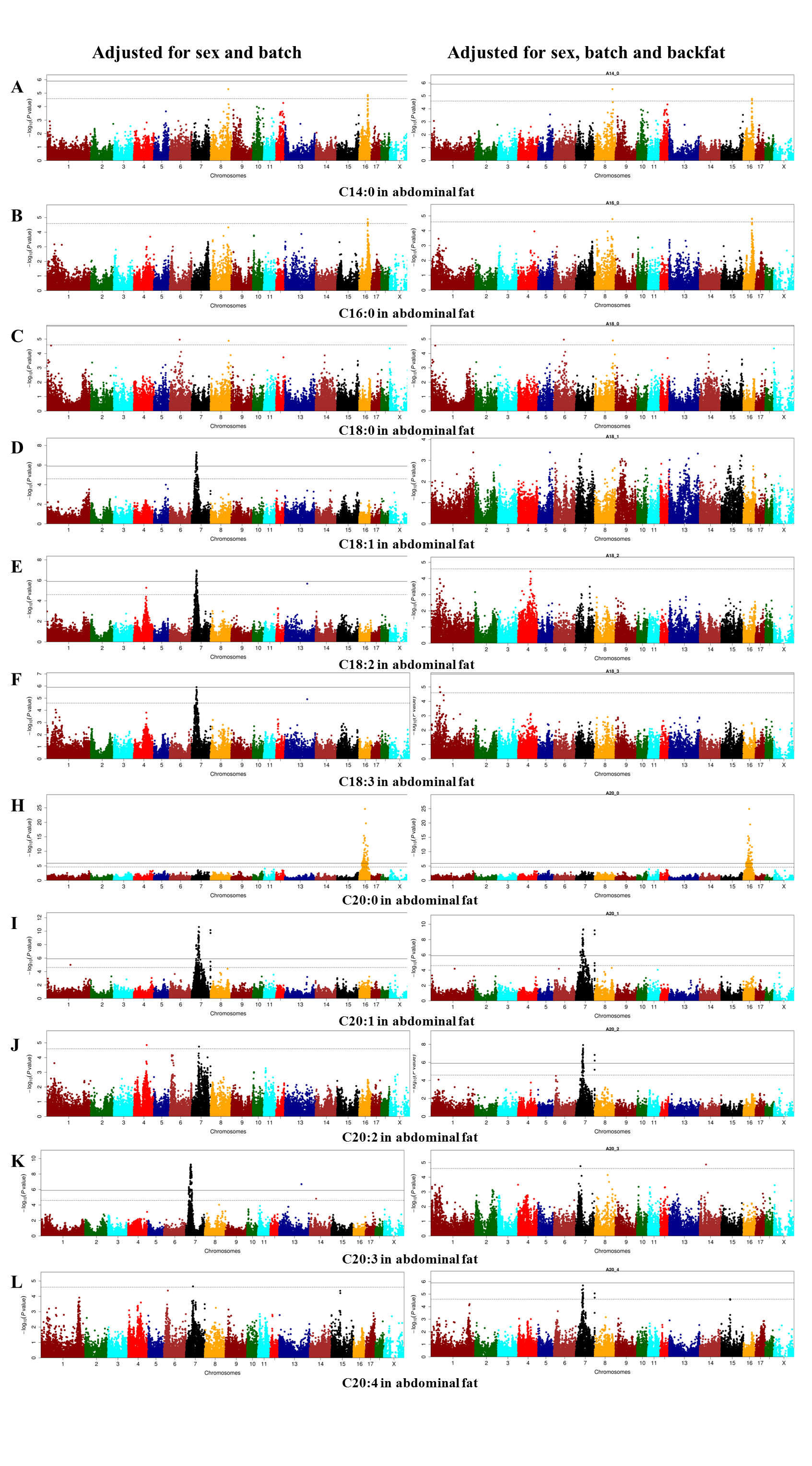

Supplement: Figure S2 — Comparison of the first round of GWAS results for fatty acid composition in abdominal fat before and after adjusting for backfat thickness in F2 animals. Fatty acid traits are shown under figures in each panel. The panels at the left side show the results from the model without a covariate of backfat thickness, and the right panels represent the results after adjusting for backfat thickness. (TIF) [file pone.0065554.s002.tif]

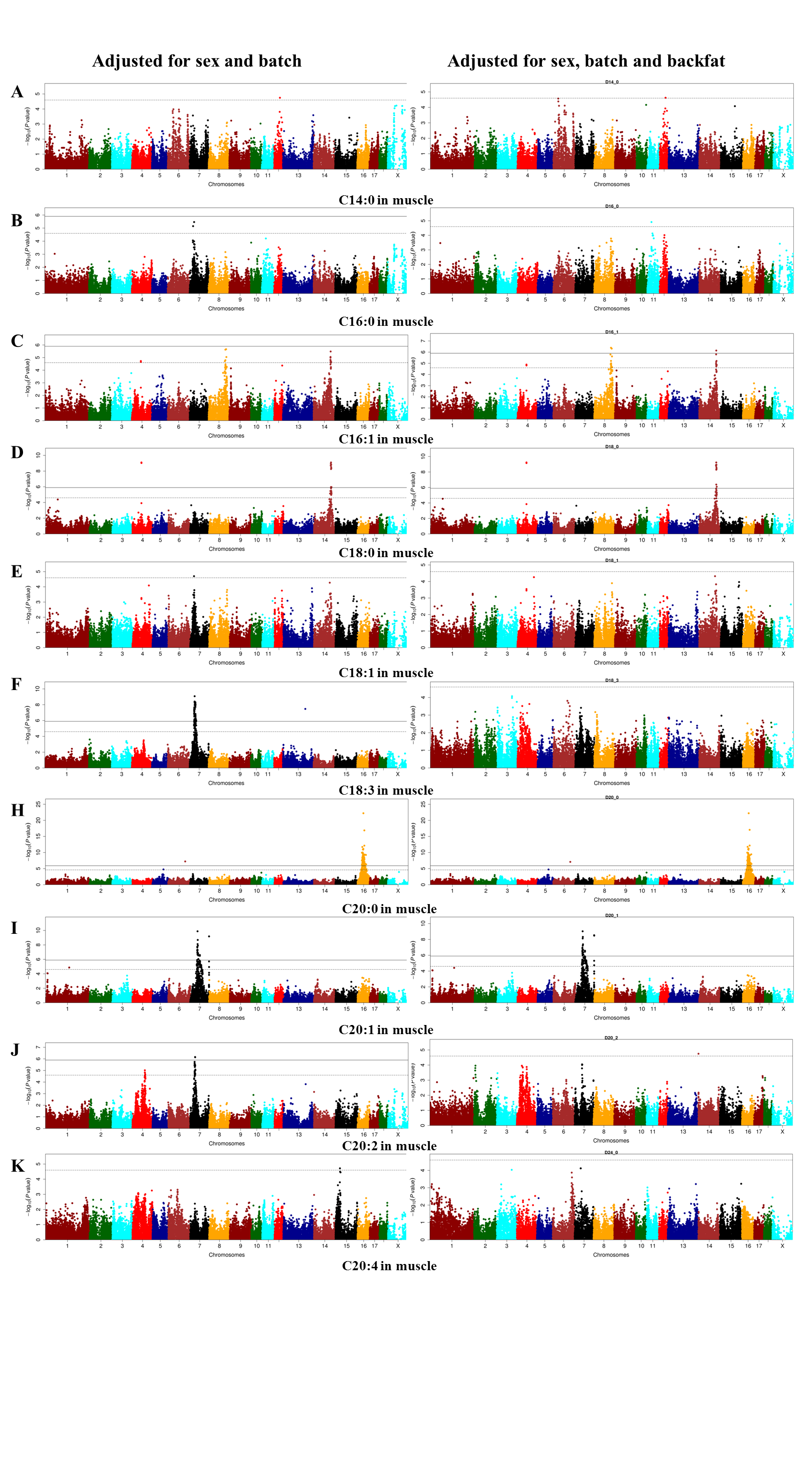

Supplement: Figure S3 — Comparison of the first round of GWAS results for muscle fat fatty acids with or without controlling for backfat thickness in F2 animals. Traits are shown under figures in each panel. The panels at the left side show the results from the model without a covariate of backfat thickness, and the right panels represent the results after correcting for backfat thickness. (TIF) [file pone.0065554.s003.tif]

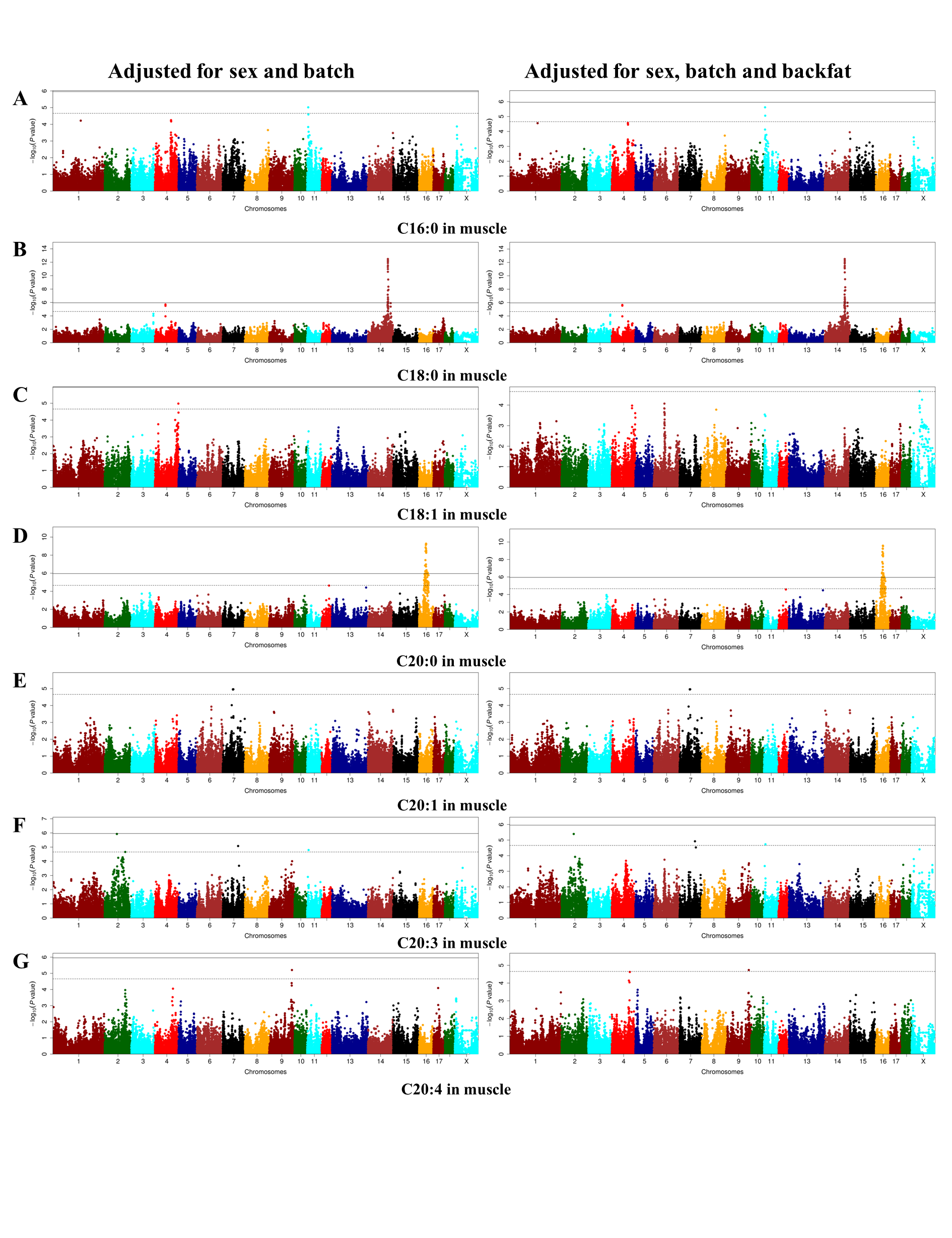

Supplement: Figure S4 — Comparison of the first round of GWAS results for muscle fat fatty acids with or without adjusting for backfat thickness in Sutai pigs. Traits are shown under figures in each panel. The panels at the left side show the results from the model without a covariate of backfat thickness, and the right panels represent the results after adjusting for backfat thickness. (TIF) [file pone.0065554.s004.tif]
