# Supplementary material for: Genome-Wide Association Analyses for Fatty Acid Composition in Porcine Muscle and Abdominal Fat Tissues
Source: PLoS One. 2013 Jun 7;8(6):e65554. doi: 10.1371/journal.pone.0065554 (PMC3676363; doi:10.1371/journal.pone.0065554)
Supplement: Table S1 — All loci surpassing the suggestive significance level for fatty acid composition identified in this study. (DOC) [file pone.0065554.s005.doc]

**Table S1** All loci surpassing the suggestive significance level for fatty acid composition identified in this study.

| Chr ^a^ | Trait | Tissue ^b^ | Population ^c^ | Top SNP | Position (bp) | *P* value | Consistence with previous QTL ^d^ | Candidate genes |
| --- | --- | --- | --- | --- | --- | --- | --- | --- |
| 1 | C20:1 | LD | F_2_ | ss131129689 | 15846521 | 1.38E-05 | Novel |  |
|  | C18:0 | LD | F_2_ | ss478939522 | 88055468 | 2.02E-06 | Novel |  |
| 2 | C20:3 | LD | Sutai | ss478939522 | 74701437 | 1.19E-06 | - |  |
| 3 | C16:1 | LD | Meta | ss131216434 | 58059120 | 2.35E-06 | - |  |
|  | C18:0 | LD | Sutai | ss107800837 | 134411189 | 1.63E-05 | - |  |
| 4 | C18:0 | LD | Meta | ss478943160 | 63841367 | 2.23E-07 | - |  |
|  | C20:2 | LD | F_2_ | ss131270860 | 88385240 | 1.01E-05 | Replicated | *ALDH9A1, HSD17B7* |
|  | C20:2 | LD | Meta | ss131270937 | 88926559 | 1.38E-05 | - |  |
|  | C18:2 | AF | F_2_ | ss131270946 | 89038458 | 1.49E-05 | Replicated |  |
|  | C20:2 | AF | F_2_ | ss120030566 | 91696280 | 1.40E-05 | Replicated |  |
|  | C18:1 | LD | Meta | ss107879085 | 123692404 | 4.21E-06 | - |  |
|  | C18:1 | LD | Sutai | ss131250986 | 142384587 | 1.05E-05 | - |  |
| 5 | C20:0 | AF | F_2_ | ss131290260 | 69369016 | 2.09E-05 | Novel |  |
|  | C16:1 | LD | Meta | ss131292052 | 75206035 | 4.46E-06 | - |  |
|  | C20:0 | LD | F_2_ | ss131292619 | 77556266 | 1.96E-07 | Replicated | *ADIPOR2, ABCD2* |
| 6 | C20:2 | AF | F_2_ | ss131317946 | 23836861 | 1.31E-05 | Novel |  |
|  | C18:0 | AF | F_2_ | ss131045334 | 72558035 | 1.09E-05 | Novel |  |
| 7 | C18:1 | LD | F_2_ | LDIAS0001336 | 27128290 | 1.99E-05 | Replicated |  |
|  | C16:0 | LD | F_2_ | ss131341918 | 31487843 | 3.46E-06 | Replicated |  |
|  | C16:0 | LD | Meta | ss131341918 | 31487843 | 1.30E-05 | - |  |
|  | C18:1 | AF | F_2_ | ss107837325 | 34803564 | 5.07E-08 | Replicated |  |
|  | C18:2 | AF | F_2_ | ss107837325 | 34803564 | 1.08E-07 | Replicated |  |
|  | C18:3 | AF | F_2_ | ss107837325 | 34803564 | 1.23E-06 | Replicated |  |
|  | C20:2 | LD | F_2_ | ss107837325 | 34803564 | 6.94E-07 | Replicated |  |
|  | C18:3 | LD | F_2_ | ss107806758 | 35177641 | 8.42E-10 | Replicated | *PPARD, HMGA1* |
|  | C20:3 | AF | F_2_ | ss131344094 | 35251345 | 5.88E-10 | Replicated |  |
|  | C20:4 | AF | F_2_ | ss131351445 | 50844200 | 2.25E-05 | Replicated |  |
|  | C20:1 | LD | F_2_ | ss131351882 | 52184508 | 1.30E-10 | Replicated |  |
|  | C20:1 | AF | F_2_ | ss131351882 | 52184508 | 2.39E-11 | Replicated | *ACSBG1* |
|  | C20:1 | LD | Meta | ss131351882 | 52184508 | 2.16E-11 | - |  |
|  | C20:1 | AF | F_2_ | ss131352160 | 52531709 | 2.24E-05 | Replicated |  |
|  | C20:2 | AF | F_2_ | ss131352216 | 52595066 | 1.77E-05 | Replicated |  |
|  | C20:1 | AF | F_2_ | ss107804785 | 53102034 | 5.92E-06 | Replicated |  |
|  | C20:1 | LD | Sutai | ss131352578 | 53371767 | 1.27E-05 | - |  |
|  | C20:1 | LD | F_2_ | ss131354957 | 56215710 | 1.87E-05 | Replicated |  |
|  | C20:1 | LD | Sutai | ss131354957 | 64593766 | 1.09E-05 | - |  |
| 8 | C16:1 | LD | F_2_ | ss131376859 | 124804112 | 2.18E-06 | Replicated | *ELOVL6, MTTP* |
|  | C14:0 | AF | F_2_ | ss107824142 | 126831850 | 5.02E-06 | Novel |  |
|  | C16:1 | LD | Meta | ss107824142 | 126831850 | 1.26E-05 | - |  |
| 9 | C20:4 | LD | Sutai | ss131407752 | 138556358 | 6.27E-06 | - | *PTGS2, PLA2G4A* |
|  | C20:4 | LD | Meta | ss131407829 | 138754600 | 2.82E-05 | - |  |
| 10 | C20:0 | LD | F_2_ | ss131041151 | 71688524 | 7.81E-06 | Novel |  |
| 11 | C16:0 | LD | Sutai | ss131445807 | 6191979 | 9.61E-06 | - |  |
| 12 | C14:0 | LD | F_2_ | ss107827572 | 41556801 | 1.79E-05 | Novel | *PCTP, ACACA* |
|  | C16:1 | LD | F_2_ | ss131468564 | 57807042 | 2.08E-05 | Novel |  |
| 14 | C20:3 | AF | F_2_ | ss107854164 | 44955016 | 2.85E-06 | Novel |  |
|  | C18:1 | LD | F_2_ | ss131498207 | 112477760 | 2.22E-05 | Novel |  |
|  | C18:1 | LD | Meta | ss131499629 | 120188229 | 1.31E-05 | - |  |
|  | C18:0 | LD | F_2_ | ss478935891 | 121305916 | 7.99E-10 | Novel | *SCD* |
|  | C18:0 | LD | Meta | ss478935891 | 121305916 | 3.76E-20 | - |  |
|  | C18:0 | LD | Sutai | ss478935891 | 121305916 | 3.29E-13 | - |  |
|  | C16:1 | LD | F_2_ | ss131499825 | 121330920 | 7.29E-07 | Novel |  |
|  | C16:1 | LD | Meta | ss131499825 | 121330920 | 7.81E-08 | - |  |
| 15 | C20:4 | LD | F_2_ | ss131569414 | 31495864 | 1.93E-05 | Novel |  |
|  | C18:1 | LD | Meta | ss478935208 | 130156404 | 1.51E-05 | - |  |
| 16 | C20:0 | LD | F_2_ | ss131535508 | 41393886 | 6.26E-23 | Replicated |  |
|  | C20:0 | AF | F_2_ | ss131535508 | 41393886 | 2.48E-25 | Replicated | *ELOVL7* |
|  | C20:0 | LD | Meta | ss131535508 | 41393886 | 2.02E-23 | - |  |
|  | C20:0 | LD | Sutai | ss131535645 | 42856916 | 5.38E-10 | - |  |
|  | C16:0 | AF | F_2_ | ss131537455 | 61060376 | 1.28E-05 | Replicated |  |

^a^ Chromosome

^b^ LD, the *longissimus dorsi* muscle. AF, abdominal fat

^c^ Meta, the loci detected by the meta-analysis.

^d^ Replicated or novel loci compared to our previously identified QTL in the F_2_ cross [8]
