# Supplementary material for: Genome-Wide Association Analyses for Fatty Acid Composition in Porcine Muscle and Abdominal Fat Tissues
Source: PLoS One. 2013 Jun 7;8(6):e65554. doi: 10.1371/journal.pone.0065554 (PMC3676363; doi:10.1371/journal.pone.0065554)
Supplement: Table S2 — Significant SNPs for C18∶1, C18∶3, C20∶0 and C20∶1 across muscle and abdominal fat tissues in the F2 population. (DOC) [file pone.0065554.s006.doc]

Table S1 Significant SNPs for C18:1, C18:3, C20:0 and C20:1 across muscle and abdominal fat tissues in the F_2_ population.

| Trait | SNP | Chr ^a^ | Pos (bp) ^b^ | Abdominal fat | | Muscle | |
| --- | --- | --- | --- | --- | --- | --- | --- |
|  |  |  |  | *P* value ^c^ | Effect (%) ^d^ | *P* value ^c^ | Effect ^d^ |
| C18:1 | ss478936930 | 7 | 27128290 | 4.70 | -1.076 | 5.27 | -1.017 |
| C18:3 | ss131101988 | 0 | 0 | 7.56 | 0.025 | 4.72 | 0.039 |
| C18:3 | ss107879050 | 0 | 0 | 7.56 | 0.025 | 4.72 | 0.039 |
| C18:3 | ss131342382 | 0 | 0 | 7.44 | 0.025 | 4.91 | 0.039 |
| C18:3 | ss107827214 | 7 | 31778883 | 6.84 | -0.023 | 4.93 | -0.039 |
| C18:3 | ss131342073 | 7 | 31822729 | 6.94 | -0.024 | 5.02 | -0.040 |
| C18:3 | ss131066868 | 7 | 31914593 | 7.18 | -0.024 | 4.89 | -0.039 |
| C18:3 | ss107884611 | 7 | 31945954 | 7.18 | -0.024 | 4.89 | -0.039 |
| C18:3 | ss478944605 | 7 | 31976416 | 7.18 | -0.024 | 4.89 | -0.039 |
| C18:3 | ss131342155 | 7 | 31989509 | 7.24 | -0.025 | 4.95 | -0.040 |
| C18:3 | ss131342171 | 7 | 32016844 | 7.44 | -0.025 | 4.94 | -0.039 |
| C18:3 | ss107860815 | 7 | 32851505 | 6.58 | 0.022 | 5.17 | 0.039 |
| C18:3 | ss131342392 | 7 | 32858692 | 7.62 | -0.025 | 5.17 | -0.040 |
| C18:3 | ss131342502 | 7 | 32997273 | 7.53 | 0.025 | 5.21 | 0.041 |
| C18:3 | ss131342510 | 7 | 33017627 | 7.33 | 0.024 | 4.61 | 0.036 |
| C18:3 | ss131342638 | 7 | 33259683 | 7.53 | 0.025 | 5.21 | 0.041 |
| C18:3 | ss131342658 | 7 | 33299125 | 7.43 | 0.025 | 4.90 | 0.039 |
| C18:3 | ss478941595 | 7 | 33388999 | 7.50 | 0.025 | 4.96 | 0.040 |
| C18:3 | ss131343534 | 7 | 34556148 | 6.19 | 0.022 | 4.97 | 0.038 |
| C18:3 | ss131343748 | 7 | 34755602 | 8.40 | 0.027 | 5.69 | 0.043 |
| C18:3 | ss107837325 | 7 | 34803564 | 8.37 | 0.026 | 5.91 | 0.042 |
| C18:3 | ss131344142 | 7 | 35002839 | 8.33 | 0.027 | 5.62 | 0.043 |
| C18:3 | ss131344154 | 7 | 35017672 | 8.33 | 0.027 | 5.62 | 0.043 |
| C18:3 | ss131344181 | 7 | 35150544 | 8.40 | 0.027 | 5.69 | 0.043 |
| C18:3 | ss107806758 | 7 | 35177641 | 9.07 | 0.027 | 5.29 | 0.039 |
| C18:3 | ss131344094 | 7 | 35251345 | 8.33 | 0.027 | 5.62 | 0.043 |
| C18:3 | ss131344285 | 7 | 35332373 | 7.95 | 0.026 | 5.32 | 0.042 |
| C18:3 | ss131344295 | 7 | 35356274 | 7.95 | 0.026 | 5.32 | 0.042 |
| C18:3 | ss131344417 | 7 | 35530333 | 7.95 | 0.026 | 5.32 | 0.042 |
| C18:3 | ss478941601 | 7 | 35579961 | 7.95 | 0.026 | 5.32 | 0.042 |
| C18:3 | ss131344469 | 7 | 35709335 | 8.23 | 0.027 | 5.34 | 0.042 |
| C18:3 | ss131344655 | 7 | 36497507 | 8.03 | 0.026 | 5.56 | 0.043 |
| C18:3 | ss131344913 | 7 | 37231172 | 7.47 | 0.024 | 4.91 | 0.037 |
| C18:3 | ss131344940 | 7 | 37288793 | 8.01 | 0.025 | 5.47 | 0.040 |
| C18:3 | ss131345041 | 7 | 37395581 | 7.96 | 0.026 | 5.49 | 0.042 |
| C18:3 | ss131346219 | 7 | 39785801 | 7.93 | 0.026 | 4.63 | 0.038 |
| C18:3 | ss131346335 | 7 | 39906109 | 7.99 | 0.026 | 4.81 | 0.039 |
| C18:3 | ss131344757 | 13 | 1.59E+08 | 7.47 | 0.024 | 4.91 | 0.037 |
| C20:0 | ss131065691 | 0 | 0 | 8.30 | 0.034 | 5.60 | 0.027 |
| C20:0 | ss131067034 | 0 | 0 | 8.31 | 0.034 | 5.58 | 0.027 |
| C20:0 | ss107896233 | 0 | 0 | 5.70 | 0.027 | 5.29 | 0.023 |
| C20:0 | ss107826158 | 16 | 25164268 | 4.64 | -0.021 | 5.28 | -0.021 |
| C20:0 | ss478937115 | 16 | 27702949 | 4.66 | -0.022 | 6.71 | -0.025 |
| C20:0 | ss131115315 | 16 | 30956011 | 7.55 | 0.031 | 4.76 | 0.023 |
| C20:0 | ss131534355 | 16 | 31016837 | 9.95 | 0.039 | 12.91 | 0.040 |
| C20:0 | ss131534376 | 16 | 31153189 | 6.17 | 0.028 | 7.56 | 0.028 |
| C20:0 | ss107821138 | 16 | 31263203 | 5.40 | 0.035 | 7.16 | 0.037 |
| C20:0 | ss131534413 | 16 | 31425059 | 4.97 | 0.035 | 5.62 | 0.032 |
| C20:0 | ss131534444 | 16 | 32131260 | 6.30 | 0.028 | 4.70 | 0.023 |
| C20:0 | ss131534597 | 16 | 33602866 | 8.48 | -0.032 | 5.85 | -0.024 |
| C20:0 | ss107904581 | 16 | 33672880 | 11.72 | 0.038 | 15.38 | 0.040 |
| C20:0 | ss131534616 | 16 | 33735604 | 5.50 | 0.024 | 5.92 | 0.023 |
| C20:0 | ss478937368 | 16 | 33954882 | 5.67 | 0.025 | 5.75 | 0.023 |
| C20:0 | ss131119964 | 16 | 34291305 | 8.03 | 0.032 | 5.84 | 0.027 |
| C20:0 | ss131534727 | 16 | 34484442 | 6.91 | 0.037 | 8.12 | 0.035 |
| C20:0 | ss131534755 | 16 | 34715842 | 4.81 | 0.023 | 5.16 | 0.022 |
| C20:0 | ss131534938 | 16 | 35661077 | 7.69 | 0.029 | 4.95 | 0.022 |
| C20:0 | ss131535013 | 16 | 36026675 | 6.07 | 0.025 | 6.12 | 0.023 |
| C20:0 | ss131535022 | 16 | 36070773 | 6.39 | 0.027 | 8.67 | 0.029 |
| C20:0 | ss131535028 | 16 | 36082305 | 8.16 | 0.029 | 9.51 | 0.029 |
| C20:0 | ss478937455 | 16 | 36540209 | 9.99 | 0.037 | 6.86 | 0.029 |
| C20:0 | ss478937922 | 16 | 36540209 | 8.96 | 0.035 | 6.29 | 0.028 |
| C20:0 | ss107870194 | 16 | 36822941 | 6.06 | 0.036 | 8.33 | 0.039 |
| C20:0 | ss131083665 | 16 | 37072576 | 5.77 | 0.041 | 5.97 | 0.037 |
| C20:0 | ss131535182 | 16 | 37408125 | 9.73 | 0.033 | 10.41 | 0.031 |
| C20:0 | ss131535179 | 16 | 37423396 | 9.55 | 0.036 | 6.75 | 0.029 |
| C20:0 | ss131111926 | 16 | 37673844 | 10.94 | 0.046 | 14.27 | 0.047 |
| C20:0 | ss131535314 | 16 | 38745958 | 7.76 | 0.032 | 5.64 | 0.026 |
| C20:0 | ss131535326 | 16 | 38763063 | 9.50 | 0.037 | 7.05 | 0.030 |
| C20:0 | ss120023490 | 16 | 38814746 | 9.50 | 0.037 | 7.05 | 0.030 |
| C20:0 | ss131535369 | 16 | 39171047 | 9.57 | 0.036 | 7.47 | 0.031 |
| C20:0 | ss131081540 | 16 | 39349056 | 9.56 | 0.037 | 6.66 | 0.029 |
| C20:0 | ss107848526 | 16 | 39433001 | 9.41 | 0.036 | 6.72 | 0.029 |
| C20:0 | ss107867042 | 16 | 39446151 | 9.63 | 0.037 | 6.83 | 0.030 |
| C20:0 | ss131535380 | 16 | 39452720 | 9.45 | 0.036 | 6.53 | 0.029 |
| C20:0 | ss131535378 | 16 | 39468170 | 9.48 | 0.036 | 6.56 | 0.029 |
| C20:0 | ss131535405 | 16 | 39814924 | 9.51 | 0.036 | 6.44 | 0.029 |
| C20:0 | ss120023622 | 16 | 39865475 | 10.91 | 0.036 | 13.65 | 0.037 |
| C20:0 | ss107818272 | 16 | 39993955 | 7.92 | 0.033 | 5.80 | 0.027 |
| C20:0 | ss107800808 | 16 | 40317027 | 7.66 | 0.032 | 5.59 | 0.027 |
| C20:0 | ss131535465 | 16 | 40383484 | 8.67 | 0.035 | 5.80 | 0.027 |
| C20:0 | ss120023619 | 16 | 40543792 | 7.66 | 0.032 | 5.59 | 0.027 |
| C20:0 | ss107868678 | 16 | 40932270 | 11.13 | 0.046 | 14.65 | 0.048 |
| C20:0 | ss131535508 | 16 | 41393886 | 22.20 | 0.053 | 24.61 | 0.050 |
| C20:0 | ss131535519 | 16 | 41435065 | 4.94 | -0.033 | 10.84 | -0.045 |
| C20:0 | ss131071064 | 16 | 41790806 | 8.28 | 0.034 | 5.75 | 0.028 |
| C20:0 | ss131535564 | 16 | 42082484 | 8.21 | 0.034 | 5.20 | 0.026 |
| C20:0 | ss131535570 | 16 | 42123776 | 8.20 | 0.034 | 5.36 | 0.026 |
| C20:0 | ss131535582 | 16 | 42177438 | 8.10 | 0.034 | 5.41 | 0.027 |
| C20:0 | ss131535589 | 16 | 42201092 | 8.34 | 0.034 | 5.58 | 0.027 |
| C20:0 | ss131535599 | 16 | 42269129 | 8.18 | 0.034 | 5.48 | 0.027 |
| C20:0 | ss131535602 | 16 | 42280991 | 8.18 | 0.034 | 5.48 | 0.027 |
| C20:0 | ss131535663 | 16 | 43203863 | 8.06 | 0.034 | 5.50 | 0.027 |
| C20:0 | ss131535671 | 16 | 43402965 | 7.73 | 0.036 | 8.34 | 0.034 |
| C20:0 | ss131535689 | 16 | 43451462 | 8.46 | 0.034 | 5.16 | 0.025 |
| C20:0 | ss131535698 | 16 | 44390463 | 6.14 | 0.027 | 6.24 | 0.025 |
| C20:0 | ss107828951 | 16 | 44429692 | 8.52 | 0.035 | 5.60 | 0.027 |
| C20:0 | ss478943653 | 16 | 44694093 | 8.52 | 0.035 | 5.60 | 0.027 |
| C20:0 | ss120023767 | 16 | 44763969 | 9.01 | 0.035 | 6.40 | 0.029 |
| C20:0 | ss131535770 | 16 | 45112154 | 8.17 | 0.034 | 5.67 | 0.027 |
| C20:0 | ss131535767 | 16 | 45251439 | 8.17 | 0.034 | 5.67 | 0.027 |
| C20:0 | ss131535775 | 16 | 45350052 | 8.17 | 0.034 | 5.67 | 0.027 |
| C20:0 | ss131535820 | 16 | 45829750 | 8.27 | 0.034 | 5.69 | 0.027 |
| C20:0 | ss107902268 | 16 | 46558952 | 8.50 | 0.034 | 5.82 | 0.027 |
| C20:0 | ss131569672 | 16 | 46602679 | 8.27 | 0.034 | 5.69 | 0.027 |
| C20:0 | ss131033258 | 16 | 46749676 | 8.27 | 0.034 | 4.93 | 0.024 |
| C20:0 | ss131535852 | 16 | 46804222 | 8.27 | 0.034 | 5.69 | 0.027 |
| C20:0 | ss131535848 | 16 | 46868015 | 6.28 | 0.027 | 5.87 | 0.024 |
| C20:0 | ss131535861 | 16 | 46893077 | 8.11 | 0.033 | 6.40 | 0.028 |
| C20:0 | ss131535874 | 16 | 46997060 | 8.27 | 0.034 | 5.69 | 0.027 |
| C20:0 | ss131535890 | 16 | 47335214 | 8.27 | 0.034 | 5.69 | 0.027 |
| C20:0 | ss131535887 | 16 | 47361829 | 8.34 | 0.034 | 5.86 | 0.028 |
| C20:0 | ss120023850 | 16 | 47498745 | 8.25 | 0.034 | 5.70 | 0.027 |
| C20:0 | ss131535902 | 16 | 47536798 | 8.27 | 0.034 | 5.69 | 0.027 |
| C20:0 | ss131535944 | 16 | 47944374 | 8.00 | 0.038 | 9.60 | 0.037 |
| C20:0 | ss131535964 | 16 | 48130360 | 12.14 | 0.039 | 12.17 | 0.035 |
| C20:0 | ss131536009 | 16 | 48203983 | 16.88 | 0.047 | 19.65 | 0.046 |
| C20:0 | ss131536042 | 16 | 48460876 | 7.78 | 0.033 | 6.46 | 0.030 |
| C20:0 | ss131536020 | 16 | 48479419 | 6.84 | -0.028 | 5.37 | -0.023 |
| C20:0 | ss131536058 | 16 | 48725323 | 6.72 | 0.031 | 6.19 | 0.029 |
| C20:0 | ss107879295 | 16 | 48917468 | 5.04 | 0.035 | 5.59 | 0.032 |
| C20:0 | ss131569655 | 16 | 49118006 | 6.09 | 0.029 | 4.78 | 0.025 |
| C20:0 | ss131536165 | 16 | 49512115 | 6.00 | 0.029 | 4.98 | 0.025 |
| C20:0 | ss120023700 | 16 | 49563861 | 6.00 | 0.029 | 4.98 | 0.025 |
| C20:0 | ss131536204 | 16 | 49868072 | 5.96 | 0.029 | 4.71 | 0.025 |
| C20:0 | ss131536227 | 16 | 50417558 | 6.58 | 0.030 | 4.86 | 0.025 |
| C20:0 | ss131536246 | 16 | 50694016 | 5.73 | 0.028 | 4.93 | 0.025 |
| C20:0 | ss131536257 | 16 | 50786940 | 9.34 | 0.036 | 9.50 | 0.033 |
| C20:0 | ss131536272 | 16 | 50902672 | 5.73 | 0.028 | 4.93 | 0.025 |
| C20:0 | ss131536335 | 16 | 51865432 | 6.33 | 0.029 | 5.11 | 0.025 |
| C20:0 | ss107825610 | 16 | 52439349 | 5.38 | 0.025 | 5.08 | 0.022 |
| C20:0 | ss76901124 | 16 | 55330045 | 6.49 | 0.033 | 8.84 | 0.035 |
| C20:0 | ss131536672 | 16 | 56305607 | 4.74 | 0.027 | 6.42 | 0.028 |
| C20:0 | ss131537125 | 16 | 59605947 | 9.25 | 0.033 | 10.08 | 0.031 |
| C20:0 | ss131537433 | 16 | 60685405 | 4.71 | -0.020 | 5.47 | -0.020 |
| C20:0 | ss120023568 | 16 | 63725230 | 4.82 | 0.022 | 6.99 | 0.024 |
| C20:1 | ss131104038 | 0 | 0 | 4.78 | 0.063 | 4.81 | 0.080 |
| C20:1 | ss131079982 | 0 | 0 | 7.20 | -0.085 | 8.29 | -0.118 |
| C20:1 | ss131066125 | 0 | 0 | 4.87 | -0.068 | 8.61 | -0.120 |
| C20:1 | ss131070614 | 0 | 0 | 7.70 | -0.088 | 8.47 | -0.119 |
| C20:1 | ss131032768 | 0 | 0 | 5.60 | 0.076 | 5.47 | 0.096 |
| C20:1 | ss107909883 | 0 | 0 | 6.63 | -0.082 | 5.43 | -0.094 |
| C20:1 | ss131077608 | 1 | 1.7E+08 | 4.87 | -0.060 | 4.99 | -0.077 |
| C20:1 | ss131349358 | 7 | 46433394 | 5.27 | -0.067 | 8.90 | -0.114 |
| C20:1 | ss131349813 | 7 | 47119440 | 5.33 | -0.065 | 6.74 | -0.094 |
| C20:1 | ss107904896 | 7 | 47176694 | 5.12 | -0.067 | 9.00 | -0.116 |
| C20:1 | ss131350455 | 7 | 48631722 | 4.62 | -0.059 | 8.28 | -0.104 |
| C20:1 | ss131350633 | 7 | 48859582 | 5.38 | -0.072 | 9.44 | -0.126 |
| C20:1 | ss131350638 | 7 | 48905608 | 5.91 | -0.075 | 9.96 | -0.128 |
| C20:1 | ss131061662 | 7 | 48953568 | 5.38 | -0.072 | 9.44 | -0.126 |
| C20:1 | ss131350765 | 7 | 49132339 | 4.60 | -0.063 | 9.36 | -0.120 |
| C20:1 | ss107858581 | 7 | 49386672 | 4.75 | -0.067 | 9.23 | -0.124 |
| C20:1 | ss131350930 | 7 | 49532604 | 5.71 | -0.064 | 6.67 | -0.088 |
| C20:1 | ss478941654 | 7 | 49721774 | 5.67 | -0.074 | 9.01 | -0.122 |
| C20:1 | ss131351008 | 7 | 49750347 | 5.68 | -0.074 | 9.01 | -0.122 |
| C20:1 | ss107832022 | 7 | 50084357 | 7.23 | -0.085 | 8.42 | -0.118 |
| C20:1 | ss107841353 | 7 | 50174844 | 7.31 | -0.086 | 8.82 | -0.121 |
| C20:1 | ss478937334 | 7 | 50231835 | 4.67 | -0.061 | 4.93 | -0.080 |
| C20:1 | ss131351283 | 7 | 50286342 | 7.28 | -0.085 | 8.92 | -0.121 |
| C20:1 | ss131351310 | 7 | 50373438 | 7.30 | -0.086 | 8.71 | -0.120 |
| C20:1 | ss131351342 | 7 | 50492312 | 7.64 | -0.087 | 7.62 | -0.111 |
| C20:1 | ss131351358 | 7 | 50518151 | 7.76 | -0.089 | 8.86 | -0.122 |
| C20:1 | ss131351445 | 7 | 50844200 | 6.94 | -0.072 | 6.77 | -0.090 |
| C20:1 | ss107909451 | 7 | 50991091 | 6.90 | -0.082 | 8.62 | -0.119 |
| C20:1 | ss107821141 | 7 | 51233941 | 6.90 | -0.082 | 8.62 | -0.119 |
| C20:1 | ss131351553 | 7 | 51526433 | 7.02 | -0.083 | 9.05 | -0.123 |
| C20:1 | ss131351557 | 7 | 51530714 | 6.95 | -0.083 | 8.64 | -0.119 |
| C20:1 | ss120018592 | 7 | 51628878 | 7.33 | -0.086 | 9.18 | -0.124 |
| C20:1 | ss131351882 | 7 | 52184508 | 9.88 | -0.097 | 10.62 | -0.129 |
| C20:1 | ss107876725 | 7 | 52208775 | 6.65 | -0.076 | 6.95 | -0.098 |
| C20:1 | ss131352009 | 7 | 52279313 | 4.87 | -0.062 | 6.02 | -0.088 |
| C20:1 | ss131352216 | 7 | 52595066 | 5.36 | -0.065 | 6.60 | -0.092 |
| C20:1 | ss131352237 | 7 | 52631322 | 8.12 | 0.095 | 6.24 | 0.105 |
| C20:1 | ss107804785 | 7 | 53102034 | 6.74 | 0.080 | 7.81 | 0.111 |
| C20:1 | ss131099136 | 7 | 53145037 | 7.29 | 0.087 | 7.51 | 0.113 |
| C20:1 | ss131352803 | 7 | 53659203 | 8.69 | -0.082 | 9.85 | -0.111 |
| C20:1 | ss478938066 | 7 | 54529860 | 6.73 | 0.083 | 6.23 | 0.102 |
| C20:1 | ss131353855 | 7 | 54718391 | 7.90 | -0.091 | 7.00 | -0.108 |
| C20:1 | ss107885050 | 7 | 56025148 | 6.85 | -0.083 | 6.49 | -0.103 |
| C20:1 | ss131354813 | 7 | 56045243 | 6.85 | -0.083 | 6.49 | -0.103 |
| C20:1 | ss131355516 | 7 | 57576229 | 6.54 | -0.080 | 4.74 | -0.086 |
| C20:1 | ss478944575 | 7 | 57916869 | 6.05 | -0.071 | 4.75 | -0.078 |
| C20:1 | ss131357041 | 7 | 67132999 | 5.78 | 0.083 | 4.67 | 0.094 |
| C20:1 | ss131357283 | 7 | 68777145 | 6.49 | -0.082 | 5.18 | -0.093 |
| C20:1 | ss120019094 | 7 | 68854659 | 6.61 | -0.083 | 5.07 | -0.092 |
| C20:1 | ss131357522 | 7 | 70000093 | 5.60 | 0.080 | 4.89 | 0.093 |
| C20:1 | ss131357544 | 7 | 70144693 | 5.40 | 0.079 | 5.02 | 0.096 |
| C20:1 | ss131357650 | 7 | 70481910 | 5.73 | 0.083 | 4.75 | 0.095 |
| C20:1 | ss131357677 | 7 | 70600433 | 5.18 | 0.079 | 4.75 | 0.095 |
| C20:1 | ss107849758 | 7 | 70644286 | 5.33 | 0.080 | 4.96 | 0.097 |
| C20:1 | ss107849350 | 7 | 70689901 | 5.18 | 0.079 | 4.75 | 0.095 |
| C20:1 | ss107879438 | 7 | 1.34E+08 | 5.72 | -0.070 | 5.47 | -0.087 |
| C20:1 | ss107840877 | 7 | 1.34E+08 | 9.18 | -0.099 | 10.16 | -0.133 |
| C20:1 | ss131117550 | 7 | 1.35E+08 | 4.94 | 0.065 | 4.80 | 0.080 |
| C20:1 | ss131335315 | 7 | 1.35E+08 | 9.18 | -0.098 | 9.72 | -0.128 |

^a^ chromosome

^b^ SNP positions on chromosomes

^c^ association strength (-log10 *P*)

^d^ additive effects of significant SNPs
